# Acute decompensation events differentially impact the risk of nosocomial infections and short-term outcomes in patients with cirrhosis

**DOI:** 10.3389/fmed.2022.962541

**Published:** 2022-08-17

**Authors:** Xianbin Xu, Xia Yu, Kai Gong, Huilan Tu, Junjie Yao, Yan Lan, Shaoheng Ye, Haoda Weng, Yu Shi, Jifang Sheng

**Affiliations:** State Key Laboratory for Diagnosis and Treatment of Infectious Diseases, National Clinical Research Center for Infectious Diseases, Collaborative Innovation Center for Diagnosis and Treatment of Infectious Diseases, The First Affiliated Hospital, Zhejiang University School of Medicine, Hangzhou, China

**Keywords:** acute decompensation, cirrhosis, nosocomial infections, jaundice, prognosis, antibiotic prophylaxis

## Abstract

**Aims:**

This research aimed to evaluate the influence of acute decompensation (AD) events upon admission on the subsequent risk of nosocomial infections (NIs) and the synergy between AD and the following NIs on the short-term outcome.

**Methods:**

A total of 419 hospitalized individuals with cirrhosis and AD participated in the current study. Various AD events at admission and outcomes in patients with or without NIs were compared. The logistic regression and Cox proportional hazards models were designed for NIs development and liver transplant (LT)-free mortality at 28 and 90 days, respectively.

**Results:**

During hospitalization, 91 patients developed NIs. Notably, a higher proportion of patients with NIs had jaundice (52.7 vs. 30.5%; *p* < 0.001) and bacterial infections (37.4 vs. 20.7%; *p* = 0.001) at admission compared to patients without NIs, while a lower proportion suffered gastrointestinal hemorrhage (16.5 vs. 36.6%; *p* < 0.001). Multivariate analysis revealed that jaundice was independently linked with the development of NIs (OR, 2.732; 95% CI: 1.104–6.762). The 28-day (16.5 vs. 7.3%; *p* = 0.008) and 90-day (27.5 vs. 15.9%; *p* = 0.011) LT-free mortality rates of patients with NIs were significantly higher than those without NIs. According to the Cox proportional hazards model, jaundice remained an independent risk factor for 90-day death (HR, 5.775; 95% CI: 1.217–27.397). The connection between total bilirubin and 90-day mortality was nonlinear, and a 6 mg/mL threshold was proposed.

**Conclusion:**

The types of AD events differentially predispose to risk of NIs. Presenting jaundice at admission is independently associated with NIs occurrence and increased 90-day mortality of patients with NIs. Antibiotic prophylaxis may benefit this specific subset of patients.

## Introduction

Liver cirrhosis is one of the major diseases worldwide (1), and its natural history can be classically divided into compensated and symptomatic decompensated phases (2). In the latter stage, ascites, hepatic encephalopathy (HE), gastrointestinal hemorrhage (GIH), non-obstructive jaundice, or bacterial infections (BIs) herald the onset of decompensated cirrhosis and often result in hospitalization and increased liver transplant (LT)-free mortality (3). Patients with cirrhosis and acute decompensation (AD) are more vulnerable to getting BIs due to the interaction of intestinal flora imbalance, bacterial translocation, and immune dysfunction (4). BIs have been shown to independently accelerate the natural course of cirrhosis (5) and increase mortality in compensated and decompensated liver cirrhosis patients (6–8). Moreover, BIs may be a preventable cause of acute-on-chronic liver failure (ACLF), organ damage, and death (4, 9).

For individuals with cirrhosis, the incidence of nosocomial infections (NIs) is an essential aspect (10). In a large, prospective, multicenter cohort study, 15% of enrolled patients with cirrhosis developed NIs, and the prognosis was worse (10). In contrast, the incidence of NIs is much higher in patients with cirrhosis and AD (11, 12). A recent Europe study found that NIs account for 52.5% of BIs in all enrolled patients with decompensated cirrhosis (12). Another prospective study reported that NIs developed in 36% of patients with cirrhosis and AD on admission (11). More importantly, NIs are related to a higher risk of ACLF, severe sepsis, and 28-day mortality in patients with decompensated cirrhosis (11, 12). It is worth noting that patients with decompensated cirrhosis have different AD events, which may lead to differences in the incidence of NIs. For instance, patients with variceal bleeding or low ascitic fluid protein levels are at a significantly increased risk of developing BIs (13). However, current studies have not elucidated how decompensation events differentially predispose to the occurrence of NIs and affect the outcome of patients complicated with NIs. Hence, this study aimed to investigate the incidence of NIs in cirrhosis with differential AD events, the relationship between the type of AD at admission and the development of NIs, and the synergy between AD and subsequent NIs on the outcome.

## Materials and methods

### Patients and diagnosis

This study enrolled patients with AD from a prospective cohort of patients with cirrhosis hospitalized for any reason between February 2014 and March 2015. In patients with chronic liver disorders, cirrhosis was diagnosed using a combination of AD, radiographic, or endoscopic findings. AD was defined as the acute onset of overt ascites (grade 2 or 3), HE, GIH, BIs, or jaundice [total serum bilirubin (TB) > 5 mg/dL] within 1 month before enrollment or any combination of these conditions (2, 14, 15). Depending on the quantity of fluid, ascites was graded from 1 to 3: (i) grade 1: only identified by ultrasonography; (ii) grade 2: medium symmetric abdomen distension; (iii) grade 3: considerable abdominal distension (3, 15). The West Haven criteria were used to evaluate HE (16). The criteria for defining ACLF and organ failures were established by the European Association for the Study of the Liver (EASL)-Chronic Liver Failure Consortium (CLIF) (3, 17). The exclusion criteria included the following: (i) age less than 18; (ii) hepatocellular carcinoma; (iii) pregnancy; (iv) human immunodeficiency virus (HIV) infection; (v) severe chronic extrahepatic disease; (vi) recent use of immunosuppressant medications; and (vii) liver transplantation recipient.

The diagnostic criteria for BIs were as follows: (i) spontaneous bacterial peritonitis (SBP): polymorphonuclear cells in ascitic fluid more than or equal to 250/mm^3^ with or without a positive fluid culture (18); (ii) urinary tract infection: leukocytes > 10/high power-field in urine and positive cultures (19); (iii) pneumonia: new pulmonary infiltrate with clinical signs of infection (20); (iv) spontaneous bacteremia: positive blood cultures without a source of bacteremia; (v) secondary bacteremia: occurring within 24 h of an invasive procedure or catheter-related infection.; (vi) skin and soft tissue infections (SSTI): skin swelling, erythema, heat, and pain; (vii) cholangitis: intense right upper quadrant pain and radiological evidence; (viii) *Clostridium difficile* infection: diarrhea and positive *C. difficile* assay; (ix) spontaneous bacterial empyema (SBE): polymorphonuclear cells in pleural fluid > 500/mm3 (250/mm3 if positive culture).

### The screening and treatment of NIs

A panel of screening and confirmatory tests for bacterial infections, including leukocyte count, serum c-reactive protein (CRP), lung CT scan, urine and stool analysis, ascites fluid examination, and microbiological culture, was established. Specific tests were selected based on the presenting signs of patients and the decision of senior physicians. NIs are infections acquired during the process of receiving heath care that was not present during the time of admission. Infections identified within 48 h of hospitalization were considered nosocomial.

The specific empirical antibiotics regimen for different types of NIs was as follows (4, 21): (i) SBP or SBE: 3rd generation cephalosporin, piperacillin-tazobactam or carbapenem; (ii) pneumonia: piperacillin-tazobactam, 3rd generation cephalosporin, levofloxacin, or moxifloxacin; (iii) urinary tract infection: 3rd generation cephalosporin; (iv) SSTI: piperacillin-tazobactam, daptomycin or linezolid; (v) cholangitis: 3rd generation cephalosporin, piperacillin-tazobactam or carbapenem; (vi) *C. difficile* infection: Metronidazole or vancomycin (22). If the causative organism was identified, empirical antibiotic therapy was continued or adjusted depending on antimicrobial susceptibility testing and efficacy evaluation.

### Data collection

Data for all variables were collected at the time of admission or the first laboratory test after admission, and this was certainly before the development of NIs. Variables included demographics, etiology of cirrhosis, cirrhosis complications (ascites, HE, GIH, BIs, and jaundice), comorbidities (e.g., hypertension and diabetes), laboratory data for Model for End-Stage Liver Disease (MELD) score and CLIF-C organ failure score, antibiotic therapy within 2 weeks before enrollment, and systemic inflammation biomarkers (e.g., leukocyte count, neutrophil count, and CRP). In addition, the data concerning the site of infection and pathogenic bacterium of NIs were collected. The primary research outcome was LT-free mortality at 28 and 90 days after enrollment. Prognostic information was collected through continuous medical services or telephonic interviews.

### Statistical analysis

Statistical analyses were carried out using R (version 4.1.0) and SPSS (version 26.0). The chi-squared test was used to compare categorical data expressed as frequencies or percentages. Continuous variables with normal distribution were summarized as means and standard deviations (SDs) and compared using the Student's *t*-test. The non-normally continuous variables were shown as medians with interquartile ranges and compared using the Mann–Whitney U or Kruskal Wallis tests. The logistic regression model was used to detect possible correlations between cirrhosis complications and NI development. The Box-Tidwell test confirmed the assumption of linearity in the logit for continuous variables. Multicollinearity was assessed by checking the Variance Inflation Factor on a multiple regression model with the same variables. Cox proportional hazard regression was performed to identify risk variables related to 28- and 90-day LT-free mortality in enrolled patients who developed NIs. The assumption of proportional hazard models was validated by correlation studies between the weighted Schoenfeld residuals and incident timings. The Kaplan-Meier method was used to estimate the mortality of patients and subgroups. The associations between levels of serum TB and 90-day mortality of cirrhotic patients with AD and NIs were assessed on a continuous scale using restricted cubic spline curves based on the Cox proportional hazards model. The two-sided significance level was set at 0.05.

## Results

**Figure 1:**
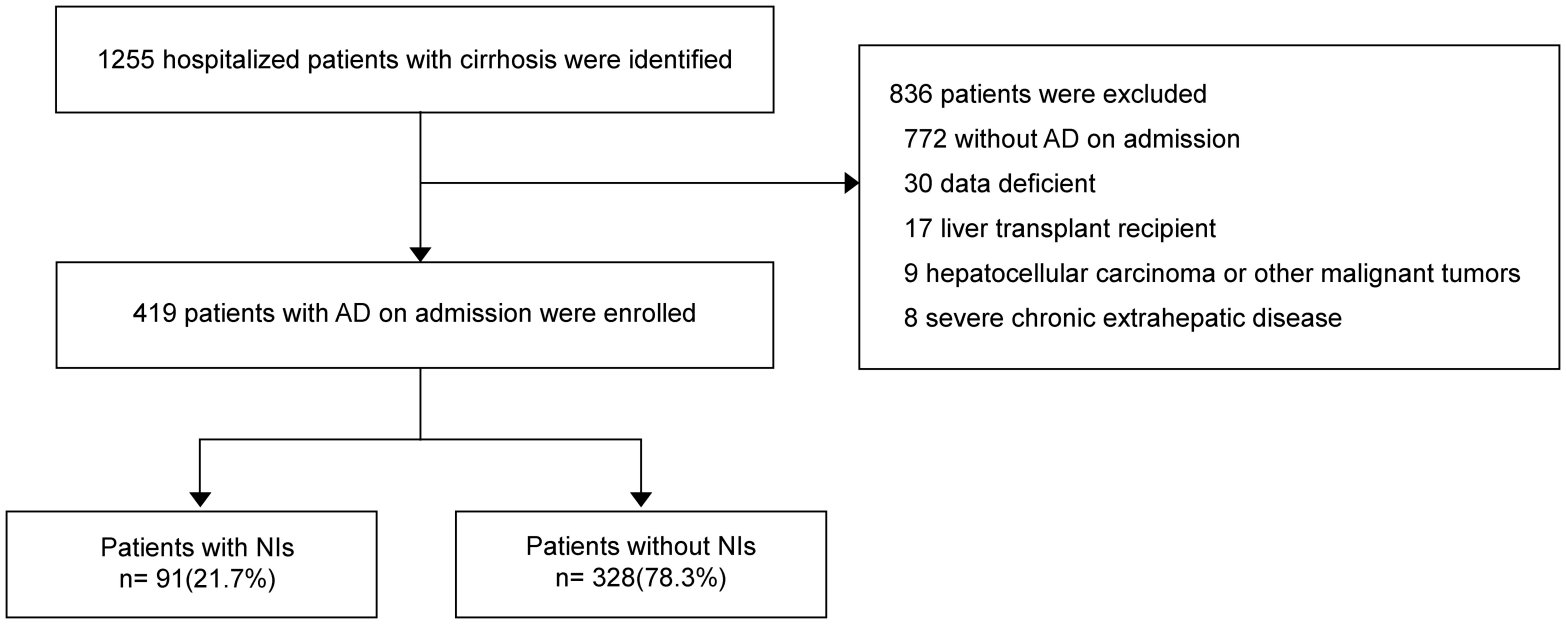
Flow chart for patient selection.

Between February 2014 and March 2015, 1248 hospitalized patients with cirrhosis were identified (Figure 1). The number of enrolled patients with AD on admission was 419 (33.4%), and 829 (66.6%) patients were excluded. Out of total patients, 91 (21.7%) developed nosocomial infections (NIs) during hospitalization.

### Clinical characteristics of enrolled patients

Patients with and without NIs had similar demographic data, etiology of cirrhosis, and comorbidities as described in Table 1. However, patients with NIs showed significantly higher levels of systemic inflammatory markers [leukocyte count, neutrophil count, and serum c-reactive protein (CRP)], international standard ratio (INR), and total serum bilirubin (TB) (Table 1). The remaining laboratory values on admission, including platelet count, hemoglobin, serum albumin, alanine aminotransferase, serum creatinine, serum sodium, and blood ammonia, were similar between the two groups. Notably, antibiotic medication within 2 weeks before enrollment (30.8 vs. 13.7%, respectively, *p* < 0.001) and ACLF on admission (22.0 vs. 13.7%, respectively, *p* < 0.05) were considerably more frequent in patients with NIs than in patients without NIs. As expected, patients who acquired NIs while hospitalized had significantly higher 28-day (16.5 vs. 7.3%, respectively, *p* < 0.05) and 90-day (27.5 vs. 15.9%, respectively, *p* < 0.05) LT-free mortality rates than patients who did not develop NIs, as patients with NIs had higher prognosis ratings, including MELD score, MELD-Na score, and CLIF-C organ failure score (*p* < 0.05).

**Table 1:** Baseline characteristics of the enrolled patients with and without NIs.

**Variables**	**Total (*n* = 419)**	**Patients with NIs (*n* = 91)**	**Patients without NIs (*n* = 328)**	***P-*value**
**Demographic data**				
Age, year, mean ± SD	54.12 ± 12.36	53.95 ± 13.10	54.17 ± 12.17	0.878
Male sex, n (%)	323 (77.1)	72 (79.1)	251 (76.5)	0.602
**Etiology of cirrhosis, n (%)**				
HBV	257 (61.3)	51 (56.0)	206 (62.8)	0.241
Alcohol	79 (18.9)	17 (18.7)	62 (18.9)	0.962
HBV & Alcohol	3 (0.7)	2 (2.2)	1 (0.3)	0.120
Others	80 (19.1)	21 (23.1)	59 (18.0)	0.274
**Comorbidities, n (%)**				
Hypertension	63 (15.0)	12 (13.2)	51 (15.5)	0.577
Diabetes	44 (10.5)	10 (11.0)	34 (10.4)	0.864
Others	35 (8.4)	8 (8.8)	27 (8.2)	0.864
**Laboratory data**				
*Biomarkers of systemic inflammation, median (IQR)*				
Leukocyte count, x10^9^/L	4.65 (2.90–7.20)	6.20 (3.75–10.43)	4.25 (2.80–6.60)	<0.001
Neutrophil count, x10^9^/L	2.90 (1.76–4.77)	4.24 (2.40–7.32)	2.60 (1.72–4.14)	<0.001
Serum CRP, mg/L	10.00 (4.10–19.08)	15.00 (8.10–29.80)	8.00 (3.40–17.45)	<0.001
Platelet count, x10^9^/L, median (IQR)	69.50 (43.00–111.25)	75.50 (50.25–133.00)	68.00 (43.00–104.00)	0.162
HB, g/L, median (IQR)	96.00 (78.00–117.00)	100.00 (83.75–119.00)	95.00 (75.25–116.00)	0.110
ALB, g/L, mean ± SD	29.38 ± 5.46	28.59 ± 5.98	29.60 ± 5.29	0.118
ALT, U/L, median (IQR)	28.00 (18.00–52.00)	29.00 (18.00–66.00)	28.00 (18.00–50.00)	0.292
TB, mg/dL, median (IQR)	2.46 (1.17–9.88)	5.91 (1.52–16.02)	2.16 (1.17–8.17)	0.002
Cr, mg/dL, median (IQR)	0.81 (0.67–1.06)	0.84 (0.72–1.12)	0.81 (0.67–1.05)	0.278
Serum sodium, mmol/L, mean ± SD	138.44 ± 5.04	138.38 ± 7.01	138.46 ± 4.35	0.924
Blood ammonia, μmol/L, median (IQR)	52.00 (33.50–78.00)	47.00 (29.00–74.50)	55.00 (34.00–78.00)	0.300
INR, median (IQR)	1.34 (1.19–1.65)	1.57 (1.86–2.38)	1.32 (1.18–1.61)	0.001
**Recent antibiotic usage** ^†^ **, n (%)**	73 (17.4)	28 (30.8)	45 (13.7)	<0.001
**ACLF, n (%)**	62 (14.8)	20 (22.0)	42 (12.8)	0.029
**Prognostic scores, mean** **±SD**				
MELD score	15.43 ± 7.90	17.67 ± 8.49	14.81 ± 7.62	0.004
MELD-Na score	16.60 ± 9.28	19.33 ± 10.08	15.84 ± 8.92	0.003
CLIF-C organ failure score	7.05 ± 1.38	7.47 ± 1.64	6.93 ± 1.27	0.004
**LT-free Mortality from enrollment, n (%)**				
28-day	39 (9.3)	15 (16.5)	24 (7.3)	0.008
90-day	77 (18.4)	25 (27.5)	52 (15.9)	0.011

† *Antibiotic therapy within 2 weeks before enrollment*.

*NIs, nosocomial infections; HBV, Hepatitis B virus; CRP, C-reactive protein; ALT, alanine aminotransferase; TB, total bilirubin; INR, international standard ratio; ACLF, acute-on-chronic liver failure; MELD score, Model for End-Stage Liver Disease score; CLIF-C: Chronic Liver Failure Consortium*.

### Profile of AD events at admission

**Figure 2:**
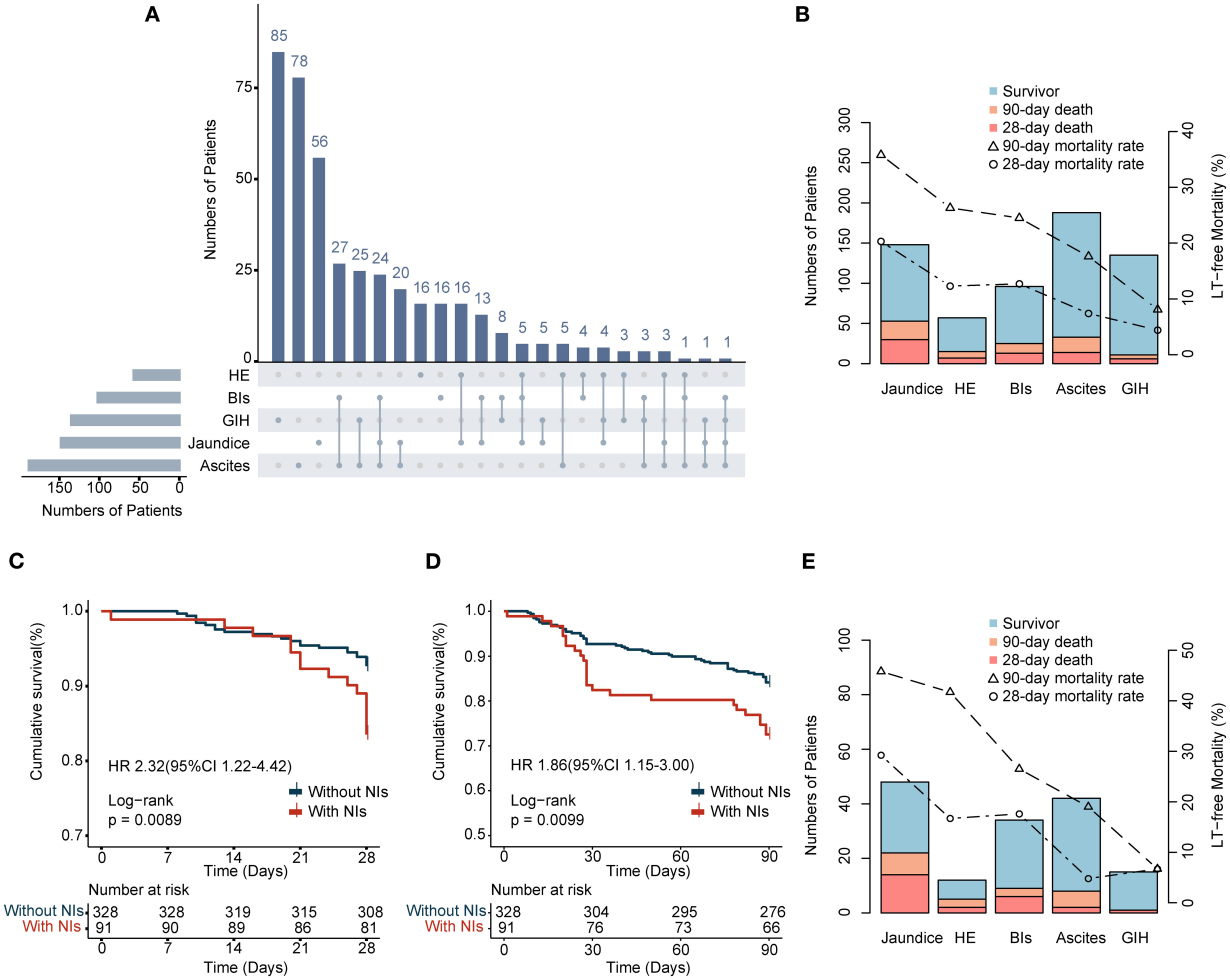
**(A)** Stacked column chart depicting combinations of AD events in enrolled patients. **(B,E)** Pareto charts showing the characteristics of AD events in two cohorts and the events-specific LT-free mortality. **(C,D)** 28- and 90-day survival curves of enrolled patients with or without NIs.

Among the 419 patients with AD, overt ascites (44.4%) was the most common AD event, followed by jaundice (35.3%) and GIH (32.3%) (Table 2). However, among those patients who acquired NIs during hospitalization, the most common event upon admission was jaundice (52.7%), followed by overt ascites (44.0%) and BIs (37.4%). The order in patients without NIs is overt ascites (44.5%), GIH (36.6%), and jaundice (30.5%). More importantly, jaundice (52.7 vs. 30.5%, *p* < 0.001) and BIs (37.4 vs. 20.7%, respectively, *p* < 0.05) occurred more frequently in patients with NIs than in those without NIs. In comparison, the incidence rate of GIH was significantly lower in patients who developed NIs (16.5 vs. 36.6%, respectively, *p* < 0.001). Regarding the number of events, the frequency of one or two complications was similar between the groups (Table 2). Patients with NIs had a higher percentage of individuals with three complications (17.6 vs. 7.6%, respectively, *p* < 0.05), but almost none had four or more complications. Details of patients with combined AD events are shown in Figure 2A and Table 2. The prevalence of specific AD events was statistically different between the groups. Notably, patients with jaundice or BIs at admission had a significantly greater incidence of NIs, while patients with GIH had a lower incidence of NIs.

**Table 2:** Prevalence of AD events in enrolled patients with and without NIs.

**Variables**	**Total (*n* = 419)**	**Patients with NIs** ** (*n* = 91)**	**Patients without NIs (*n* = 328)**	***P*-value**
**AD events of cirrhosis, n (%)**
BIs	102 (24.3)	34 (37.4)	68 (20.7)	0.001
Overt ascites	186 (44.4)	40 (44.0)	146 (44.5)	0.925
Grade 2	172 (41.1)	41 (45.1)	131 (39.9)	0.300
Grade 3	16 (3.8)	1 (1.1)	15 (4.6)	
Jaundice	148 (35.3)	48 (52.7)	100 (30.5)	<0.001
GIH	135 (32.2)	15 (16.5)	120 (36.6)	<0.001
HE	57 (13.6)	12 (13.2)	45 (13.7)	0.896
Grade I	42 (10.0)	10 (11.0)	33 (10.1)	0.979
Grade II	11 (2.6)	2 (2.2)	9 (2.7)	
Grade III~IV	3 (0.7)	0 (0)	3 (0.9)	
**Number of AD events, n (%)**
One	251 (59.9)	47 (51.6)	204 (62.2)	0.029
Two	126 (30.1)	28 (30.8)	98 (29.9)	
Three	41 (9.8)	16 (17.6)	25 (7.6) ^†^	
≥Four	1 (0.2)	0 (0)	1 (0.3)	
**Multiple AD events, n (%)**
Only GIH	85 (20.3)	8 (8.8)	77 (23.5) ^†^	0.001
Only overt ascites	78 (18.6)	12 (13.2)	66 (20.1)	
Only jaundice	56 (13.4)	18 (19.8)	38 (11.6) ^†^	
Overt ascites and BIs	27 (6.4)	7 (7.7)	20 (6.1)	
Overt ascites and GIH	25 (6.0)	3 (3.3)	22 (6.7)	
Overt ascites and jaundice and GIH	24 (5.7)	10 (11.0)	14 (4.3) ^†^	
Overt ascites and jaundice	20 (4.8)	7 (7.7)	13 (4.0)	
Only HE	16 (3.8)	3 (3.3)	13 (4.0)	
Only BIs	16 (3.8)	6 (6.6)	10 (3.0)	
Jaundice and HE	16 (3.8)	2 (2.2)	14 (4.3)	
Jaundice and BIs	13 (3.1)	6 (6.6)	7 (2.1) ^†^	
Others	43 (10.3)	9 (9.9)	34 (10.4)	

† *Significantly different from the group with NIs and group without NIs*.

*AD, acute decompensation; NIs, nosocomial infections; BIs, bacterial infections; HE, hepatic encephalopathy; GIH, gastrointestinal hemorrhage*.

### Types of NIs and isolated bacteria

As shown in Table 3, the most common type of bacterial infections in the 91 patients who developed NIs during hospitalization was SBP (39.6%), followed by pneumonia (38.4%), spontaneous or secondary bacteremia (6.6%), cholangitis (5.5%), *C. difficile* infection (4.4%), spontaneous bacterial empyema (SBE) (3.3%), and skin and soft tissue infection (SSTI) (2.2%). A total of 18 pathogens were identified in these patients. *Klebsiella pneumoniae* was the most frequently isolated bacterium (27.8%), followed by *C. difficile* (22.2%) and *Acinetobacter baumannii* (11.1%).

**Table 3:** Types of nosocomial infection and isolated bacteria.

**Variables**	**Frequency, n (%)**
**Types of infection (*****n*** **=** **91)**	***n*** **=** **91**
SBP	36 (39.6)
Pneumonia	35 (38.4)
Spontaneous or secondary bacteremia	6 (6.6)
Cholangitis	5 (5.5)
*Clostridium difficile* infection	4 (4.4)
SBE	3 (3.3)
SSTI	2 (2.2)
**Isolated bacteria**	***n*** **=** **22**
*Klebsiella pneumoniae*	5 (22.7)
*Clostridium difficile*	4 (18.2)
*Acinetobacter baumannii*	2 (9.1)
*Candida albicans*	2 (9.1)
*Candida krusei*	1 (4.5)
*Enterococcus faecium*	1 (4.5)
*Aeromonas hydrophila*	1 (4.5)
*Escherichia coli*	1 (4.5)
*Pseudomonas aeruginosa*	1 (4.5)
*Stenotrophomonas maltophilia*	1 (4.5)
*Streptococcus pluranimalium*	1 (4.5)
*Vibrio vulnificus*	1 (4.5)

*SBP, spontaneous bacterial peritonitis; SBE; spontaneous bacterial empyema; SSTI; skin and soft tissue infections*.

### Risk factors of NIs

Supplementary Table S1 shows variables associated with the occurrence of NIs in the univariate analysis. BIs and jaundice were linked to the occurrence of NIs in all types of AD events, while GIH was linked to a lower incidence of NIs. Furthermore, patients with three kinds of events simultaneously were more likely to develop NIs than those with only one [odds ratio (OR), 2.778; 95% CI: 1.375–5.611; *p* = 0.004] or two complications (OR, 2.240; 95% CI: 1.053–4.766; *p* = 0.036). In terms of laboratory tests, neutrophil count and serum CRP levels were related to the NIs development. In addition, other potential risk factors include antibiotics usage, ACLF, and MELD score. The ALCF and MELD scores were excluded from multivariate analysis because specific laboratory data were calculated in these prognostic scores. In the multivariate analysis, only jaundice (OR, 2.732; 95% CI: 1.104–6.762; *p* = 0.030), neutrophil count (OR, 1.080; 95% CI: 1.004–1.163; *P* = 0.039), and antibiotics usage within 2 weeks before enrollment (OR, 2.095; 95%CI: 1.127–3.896; *p* = 0.019) were independent predictors of NIs (Table 4).

**Table 4:** Multivariate analysis of the risk factors for the development of NIs in patients with cirrhosis and AD and short-term outcome in cirrhotic patients with AD and NIs.

**Variables**	**Risk factors for NIs**		**Risk factors for 28-day mortality**		**Risk factors for 90-day mortality**	
	**OR (95% CI)**	***P*-value**	**HR (95% CI)**	***P*-value**	**HR (95% CI)**	***P*-value**
Jaundice vs. non-jaundice	2.732 (1.104–6.762)	0.030	-	0.125	5.775 (1.217–27.397)	0.027
Neutrophil count	1.080 (1.004–1.163)	0.039	1.159 (1.077–1.248)	<0.001	1.115 (1.037–1.199)	0.003
Recent antibiotic usage^†^	2.095 (1.127–3.896)	0.019	-	-	-	-
INR	**-**	**-**	6.948 (2.602–18.555)	<0.001	3.409 (1.439–8.073)	0.005

† *Antibiotic therapy within 2 weeks before enrollment*.

*The binary logistic regression model was carried out to identify the potential links between complications of cirrhosis and NI development. Cox proportional hazard regression was used to identify risk factors associated with 28- and 90-day LT-free mortality in enrolled patients who developed NIs. Variables statistically significant at p < 0.10 on univariate analysis were applied to final models*.

*AD, acute decompensation; NIs, nosocomial infections; INR, international standard ratio*.

### Overall and events-specific LT-free mortality

Overall, 39 (9.3%) and 77 (18.4%) deaths occurred within 28 and 90 days, respectively. Among all types of cirrhosis complications, jaundice resulted in the highest LT-free mortality at 28 and 90 days (20.3 and 35.8%, respectively), followed by HE (12.3 and 26.3%), BIs (12.7 and 24.5), ascites (7.4 and 17.6%), and GIH (4.4 and 8.1%) (Figure 2B). One essential aspect is to determine the influence of NIs on prognosis. Our findings confirmed that LT-free mortality was higher in the NIs group than in the non-NIs group, reaching 16.5% at 28 days and 27.5% at 90 days (Figures 2C,D and Table 1). Furthermore, patients who developed NIs with jaundice were more prone to suffer poorer outcomes than others (28-day, 29.2%; 90-day 45.8%; Figure 2E). Moreover, patients with GIH were associated with the lowest 90-day mortality in both the overall population and the NIs group.

### Risk factors of 28- and 90-day death

**Figure 3:**
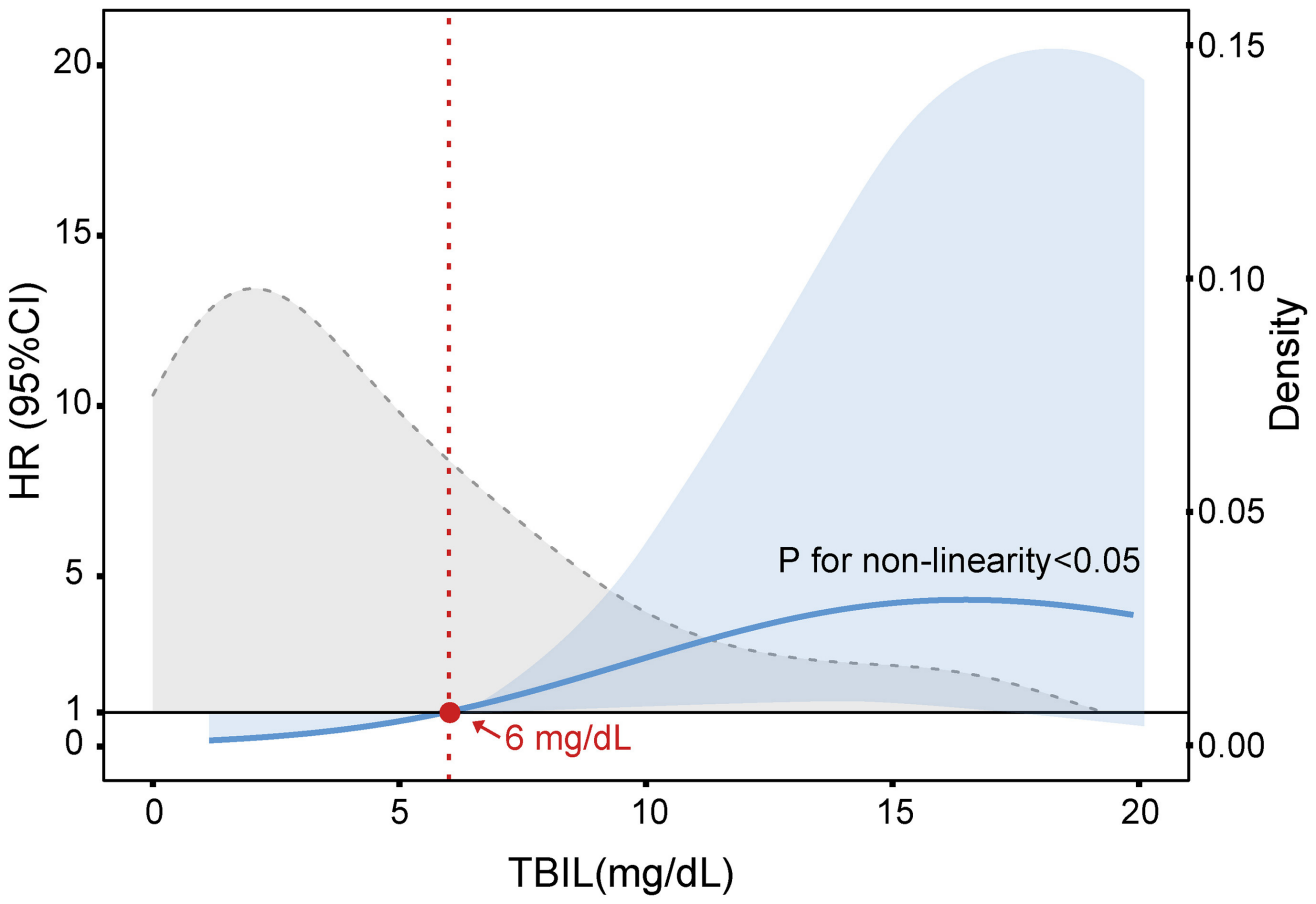
Multivariable adjusted hazard ratios (HR) for 90-day LT-free mortality according to the levels of serum total bilirubin (TB) on a continuous scale in cirrhotic patients with AD and NIs. The blue line is multivariable adjusted HR, with light blue shaded area represent 95% CI. Dashed gray curve shows the fraction of population with different TB levels. Serum TB 6 mg/dL was selected as the reference level. Analyses were adjusted for overt ascites, neutrophil count, ALT, and INR at baseline.

Next, we explored the risk factors for short-term outcomes in patients who developed NIs. The results of the univariate analysis are presented in Supplementary Table S2. Multivariate analysis revealed neutrophil count [HR 1.159; 95% CI: 1.077–1.248; *p* < 0.001] and INR (HR 6.948; 95% CI: 2.602–18.555; *p* < 0.001) were independently associated with the 28-day death (Table 4). The independent risk factors for 90-day death were jaundice (HR 5.775; 95% CI: 1.217–27.397; *p* = 0.027), neutrophil count (HR 1.115; 95% CI: 1.037–1.199; *p* = 0.003) and, INR (HR 3.409; 95% CI: 1.439–8.073; *p* = 0.005). Jaundice is generally defined as the TB > 5 mg/dL. To determine a TB threshold beyond which jaundice impacted the outcome, we used a restricted cubic spline to flexibly model and visualize the relationship between levels of TB and the probability of 90-day death on a continuous scale (Figure 3). The multivariable-adjusted hazard ratio was below the reference line (at a hazard ratio of 1.0) until around 6 mg/dL of serum TB, representing a breaking point for risk of death in patients with NIs.

## Discussion

There is a need to understand how AD events differentially impact the risk of NIs and short-term outcomes in patients with cirrhosis. In the present study, we investigated the epidemiological and microbiological features of NIs in patients with cirrhosis and AD and the relationship between NIs and cirrhosis complications. In this study, the prevalence of NIs in patients with cirrhosis and AD was 21.7%, and SBP was the most common infection. These results were consistent with previously published works (10, 23, 24). The isolated bacteria correspond to the type of infection. It has been suggested that *Enterobacteriaceae* were the most common gram-negative bacteria that cause SBP in patients with cirrhosis, including *Escherichia coli* and *Klebsiella pneumoniae* (23, 24). A worldwide study showed that these two bacteria were the most frequently isolated bacteria in cirrhotic patients with infection (24). However, *Klebsiella pneumoniae* was the most commonly isolated bacteria in our study. This difference can be due to the epidemiological characteristics of each region. It is noteworthy that the results of incidences for *Escherichia coli* and *Klebsiella pneumonia* infections in Asia were close to each other in the first infection (25 and 24%, respectively) (24). In addition, *Klebsiella pneumonia* was more common in the second infection than *Escherichia coli* (19 and 14%, respectively) (24). More importantly, the report of NIs surveillance in our center found that the prevalence of *Klebsiella pneumonia* infection was roughly 17%, ranking first (data not provided).

Among all types of AD events of cirrhosis, jaundice was the most common complication in patients with NIs on admission (Table 2). Specifically, the median level of serum TB in patients with NIs was 5.91 mg/dL, statistically higher than 2.16 mg/dL in patients without NIs. This result is in accordance with earlier published studies (10). Further, an earlier report by Field et al. showed that patients in the surgical intensive care unit with serum TB >3 mg/dL had a 3-fold higher risk of infection than patients with serum TB ≤ 3 mg/dL (25). Corroborating this report, our results also demonstrate that jaundice was not only an independent risk factor for developing NIs but also a predictor of 90-day mortality in cirrhotic patients with AD and NIs. However, the underlying mechanism by which jaundice increases the risk of BIs has not been clarified.

In recent years, growing evidence has gradually revealed the potential immunomodulatory effects of bilirubin *in vitro* and *in vivo*, which may help us delve deeper into the above issue (26–29). Liu et al. reported that bilirubin significantly suppressed antigen-specific and polyclonal T cell responses through various mechanisms, including inhibition of costimulatory molecule activities, interference with immune transcription factor activation, and suppression of the expression of class II Major Histocompatibility Complex molecules in Antigen Presenting Cells (26). Similarly, Khan et al. demonstrated that unconjugated bilirubin not only resulted in DNA strand breaks and oxidative stress in lymphocytes but induced apoptosis of various immune cells, including mouse splenic CD4^+^ T cells, CD19^+^ B cells, peritoneal exudates cells, and human peripheral blood mononuclear cells (27). For macrophage, early studies have confirmed that bilirubin may affect the expression of Fc receptors by altering the lipid environment of the cytoplasmic membrane (28). Besides, bilirubin has also been proven to inhibit the vascular cell adhesion molecule-1-dependent lymphocyte migration and reduce airway inflammation in a murine asthma model (29). These findings revealed the specific mechanism by which bilirubin inhibits the function of crucial immune cells involved in innate and adaptive immunity. Accordingly, we hypothesized that bilirubin's immunosuppressive effects might result in organisms being more susceptible to pathogens. Indeed, Khan et al. have confirmed that bilirubin pretreatment increased the susceptibility of mice to develop BIs and significantly reduced the median survival time of mice with infection (27). These findings were thought to have close relevance to bilirubin promoting the release of serum pro-inflammatory cytokines (TNF-α, IL-1β, and IL-6) and inhibiting various immune cell responses (27).

Clinically, once the serum TB level is higher than 2.5–3 mg/dL, patients will show signs of jaundice (yellowing of skin, sclera, and mucous membranes) (30). As mentioned, a study divided patients into high or low bilirubin groups with 3 mg/dl as the threshold level and revealed that the risk of infection was significantly increased in the high bilirubin group (25). Furthermore, a recent study by Patel et al. demonstrated that serum bilirubin levels are closely related to the increased risk of death in patients with severe sepsis and septic shock (31). In our study, we defined jaundice as TB > 5 mg/dL according to the guideline proposed by the Asian Pacific Association for Liver Research (APASL) (32). Our results indicated that the risk of NIs in patients with jaundice was significantly increased and is associated with significantly greater LT-free mortality. At present, however, no research has clearly proposed the threshold for predicting the outcomes of patients with NIs and cirrhosis based on serum bilirubin. Based on the restricted cubic spline model, we first demonstrated that serum TB at ~6 mg/mL was a threshold for predicting 90-day death in individuals with cirrhosis and NIs (Figure 3). This finding provides a theoretical basis for establishing a more reasonable and personalized therapeutic schedule, especially for applying the anti-infective agents. However, since our study is a single-center and retrospective cohort with a small sample size, additional prospective and multicenter studies are needed to confirm these conclusions fully.

Interestingly, our data indicate that patients with GIH had a low incidence of NIs, which is inconsistent with the previous studies (23). Past research has demonstrated that the presence of BIs is an independent predictor of failure to control bleeding and death (33). Clinical practice guidelines recommended that empirical antibiotic therapy should be started as soon as GIH is suspected (3). Therefore, the reason for this discrepancy is thought initiatively be associated with antibiotic prophylaxis in our center. Nevertheless, the remarkable thing is that empiric antibiotic treatment is not routinely used in patients with jaundice, which got us thinking: can antibiotic prophylaxis reduce the risk of NIs and short-term mortality in this subset of patients?. The pity is that no randomized controlled trial or multicenter retrospective study has investigated this issue so far.

Furthermore, the study provided evidence for the “immunoparesis hypothesis,” according to which BIs are a complication of immunoparesis to limit the vigorous proinflammatory response (34, 35). In our study, patients who developed NIs during hospitalization had significantly higher levels of certain substances regarded as the biomarkers of systemic inflammation, such as leukocyte count, neutrophil count, and serum CRP on admission compared to those without NIs. Utilizing Cox proportional hazard and binary logistic regression models, we confirmed that neutrophil count was an independent predictor for NIs acquisition as well as an independent risk factor of 28- and 90-day mortality in cirrhotic patients with AD and NIs. These findings supported previous studies highlighting the role of systemic inflammation in disease progression (34, 36, 37).

Notably, our findings showed that patients who received antibiotic therapy within 2 weeks before enrollment had approximately 2-fold higher risk of NIs compared to those without antibiotic therapy. The use of antibiotics before admission is typically indicative of concomitant infections. Previous studies have found significantly higher prevalence of secondary infections in cirrhotic patients with first infection, which was considered to be related to the immunosuppression in severely infected patients (20). At present, it is believed that sepsis can lead to immunosuppression characterized by lymphocyte apoptosis, which is clearly related to the susceptibility to NIs. (38, 39). Besides, antibiotic overuse and failure of antibiotic strategies are closely associated with infection in cirrhotic patients, especially infections caused by multidrug-resistant organisms in the healthcare setting (23, 40, 41). Lacking appropriate prophylactic or therapeutic antibiotics usage may deteriorate the organ damage and adverse outcomes caused by infection (41). Therefore, we suspected that the correlation between NIs and antibiotic usage revealed by our study might be related to the antibiotic issues stated above. It is now generally agreed that appropriate use of antibiotics (i.e., avoid overusing antibiotics, restrict antibiotic use population, and timely adjustment to sensitive antibiotic drugs based on a bacterial culture and sensitivity test results) and nonantibiotic prophylactic strategies (i.e., probiotics) can help reduce the incidence of drug resistance and increase the clinical curative effects (41). Consequently, we strongly recommend that prophylactic use of antibiotics should be directed at initial infections and that rational use of antibiotics should be taken more seriously.

This study has certain limitations. First, our study is a single-center cohort with a limited number of patients who developed NIs and lacking of external validity to support widespread changes in practice; thus, large and multicenter studies are required to corroborate our findings. Second, we only included variables at the time of admission, not at subsequent time points, which may introduce bias. Third, the hepatitis B virus was the major etiology of cirrhosis in this cohort, and the impact of etiology on the findings could not be ruled out. For instance, previous studies showed that the number of patients admitted due to jaundice was exceptionally high among those with hepatitis B virus-related cirrhosis (42, 43). Fourth, we only take short-term prognosis as the primary outcome, and the impact of acute decompensation events on long-term prognosis of cirrhotic patients with AD and NIs remains to be further explored.

In conclusion, we confirmed the high prevalence and negative short-term outcomes of NIs in hospitalized patients with cirrhosis and AD. Cirrhotic patients admitted for different types of AD events had a differential risk of developing NIs. To the best of our knowledge, we first revealed that presenting jaundice at admission is independently associated with NIs occurrence and increased 90-day mortality in NIs patients. Serum TB levels above ~6 mg/dL may be used to predict 90-day death in individuals with cirrhosis and NIs. Appropriate antibiotic prophylaxis may benefit this specific subset of cirrhotic patients.

## Data availability statement

The original contributions presented in the study are included in the article/Supplementary material, further inquiries can be directed to the corresponding authors.

## Ethics statement

The studies involving human participants were reviewed and approved by the Ethics Committee of the First Affiliated Hospital, School of Medicine, Zhejiang University. The patients/participants provided their written informed consent to participate in this study.

## Author contributions

XX, XY, YS, and JS designed the research study. XX, XY, KG, HT, JY, YL, SY, and HW performed the study and collected the data. XX and XY analyzed the data and wrote the manuscript. All authors have read and approved the final manuscript.

## Funding

This work was supported by grants from the Chinese National Natural Science Foundation (No. 81870425), Medical health S&T Projects of Zhejiang Province (No. 2022RC141), Fundamental Research Funds for the Central Universities (No. 2021FZZX001-41), and Scientific Research Fund of Zhejiang University (No. XY2021030).

## Conflict of interest

The authors declare that the research was conducted in the absence of any commercial or financial relationships that could be construed as a potential conflict of interest.

## Publisher's note

All claims expressed in this article are solely those of the authors and do not necessarily represent those of their affiliated organizations, or those of the publisher, the editors and the reviewers. Any product that may be evaluated in this article, or claim that may be made by its manufacturer, is not guaranteed or endorsed by the publisher.
