# Supplementary material for: Acute decompensation events differentially impact the risk of nosocomial infections and short-term outcomes in patients with cirrhosis
Source: Front Med (Lausanne). 2022 Aug 17;9:962541. doi: 10.3389/fmed.2022.962541 (PMC9428487; doi:10.3389/fmed.2022.962541)
Supplement: Supplementary file 1 [file Table_1.pdf]

Acute decompensation events differentially impact on risk of nosocomial infections and short-term outcome in patients with cirrhosis

Xianbin Xu, Xia Yu, Kai Gong, Huilan Tu, Junjie Yao, Yan Lan, Shaoheng Ye, Haoda Weng, Yu Shi, and Jifang Sheng

Supplemental Tables

Supplementary Table 1. Univariate analysis of the risk factors for the development of NIs in patients with cirrhosis and AD.

Variables	Univariate analysis	
	OR (95% CI)	*P* value
**Age≥55**	0.791 (0.496-1.260)	0.323
**Women vs. men**	1.163 (0.660-2.048)	0.602
**Diabetes**	1.068 (0.506-2.253)	0.864
**Etiology of cirrhosis**		
Hepatitis B virus	0.755 (0.472-1.209)	0.242
Alcohol	0.986 (0.544-1.787)	0.962
Hepatitis B virus & Alcohol	-	0.105
Others	1.368 (0.779-2.402)	0.276
**AD events of cirrhosis**		
BIs	2.281 (1.381-3.766)	0.001
Overt ascites	1.068 (0.670-1.703)	0.781
Grade2	1.162 (0.725-1.863)	0.532
Grade3	0.248 (0.032-1.921)	0.182
Jaundice	2.545 (1.584-4.088)	<0.001
GIH	0.342 (0.188-0.622)	<0.001
HE	0.955 (0.482-1.893)	0.896
GradeI	1.086 (0.513-2.298)	0.830
GradeII	0.796 (0.169-3.759)	0.773
GradeIII~IV	-	-
**Number of AD events**		
Two vs. One	1.240(0.733-2.099)	0.423
Three vs. One	2.778(1.375-5.611)	0.004
Three vs. Two	2.240(1.053-4.766)	0.036
≥Four vs. One/ Two/ Three	-	-
**Laboratory data**		
Neutrophil count	1.136(1.067-1.209)	<0.001

Serum CRP	1.017(1.007-1.026)	<0.001
Platelet count	1.002(0.999-1.005)	0.204
Hemoglobin	1.008(0.999-1.017)	0.088
Serum albumin	0.966(0.925-1.009)	0.118
ALT	1.000(0.998-1.002)	0.971
Serum creatinine	1.044(0.801-1.362)	0.749
Serum sodium	0.997(0.952-1.044)	0.901
Blood ammonia	0.997(0.989-1.006)	0.527
INR	1.343(0.958-1.883)	0.087
**Recent antibiotic usage†**	2.795(1.621-4.821)	<0.001
**ACLF**	1.918(1.061-3.469)	0.031
**MELD score**	1.045(1.016-1.076)	0.003

†Antibiotic therapy within two weeks before enrollment.

Statistical analysis was performed using a univariate binary logistic regression model.

Abbreviation: BIs, bacterial infections; GIH, gastrointestinal hemorrhage; HE, hepatic encephalopathy; CRP, C-reactive protein; ALT, alanine aminotransferase; TBIL, total bilirubin; INR, international standard ratio; ACLF, acute-on-chronic liver failure; MELD score, Model for End-Stage Liver Disease score.

Supplementary Table 2. Univariate analysis of the risk factors for 28-day and 90-day mortality in cirrhotic patients with AD and NIs.

Variables	Risk factors for 28-day mortality		Risk factors for 90-day mortality	
	HR (95% CI)	*P* value	HR (95% CI)	*P* value
**Age≥55**	1.497(0.543-4.128)	0.436	1.401(0.639-3.070)	0.400
**Women vs. men**	0.684(0.218-2.149)	0.515	0.786(0.314-1.968)	0.606
**Diabetes**	1.225(0.276-5.427)	0.790	0.692(0.163-2.934)	0.617
**Etiology of cirrhosis**				
Hepatitis B virus	1.231(0.438-3.458)	0.694	0.871(0.397-1.908)	0.729
Alcohol	0.651(0.147-2.883)	0.571	0.775(0.266-2.257)	0.640
Hepatitis B virus & Alcohol	-	-	-	-
Others	1.178(0.375-3.701)	0.779	1.642(0.708-3.809)	0.248
**AD events of cirrhosis**				
BIs	1.100(0.391-3.089)	0.857	0.940(0.415-2.128)	0.883
Overt ascites	0.160(0.036-0.710)	0.016	0.462(0.199-1.072)	0.072
Jaundice	14.498(1.906-110.301)	0.010	8.652(2.585-28.962)	<0.001
GIH	0.358(0.047-2.727)	0.322	0.192(0.026-1.422)	0.106
HE	1.023(0.231-4.536)	0.976	1.696(0.636-4.520)	0.291
**Number of AD events**				
Two vs. One	0.823(0.248-2.734)	0.751	0.809(0.303-2.154)	0.671
Three vs. One	1.128(0.299-4.251)	0.859	1.847(0.727-4.695)	0.197
Three vs. Two	1.370(0.307-6.122)	0.680	2.285(0.767-6.804)	0.138
**Type of infection**				
SBP	0.513(0.163-1.612)	0.253	0.533(0.222-1.276)	0.158
Pneumonia	1.944(0.705-5.362)	0.199	1.688(0.770-3.703)	0.191
Others	1.239(0.394-3.891)	0.714	1.291(0.539-3.091)	0.567
**Laboratory data**				
Neutrophil count	1.126(1.049-1.208)	0.001	1.103(1.029-1.183)	0.006
Serum CRP	0.998(0.979-1.017)	0.843	0.990(0.972-1.008)	0.268
Platelet count	0.997(0.988-1.005)	0.462	0.998(0.992-1.005)	0.624
HB	1.008(0.989-1.027)	0.392	1.009(0.995-1.024)	0.213
ALB	0.970(0.887-1.061)	0.504	0.965(0.900-1.034)	0.308
ALT	1.003(0.999-1.007)	0.149	1.004(1.001-1.007)	0.010
Cr	1.259(0.726-2.183)	0.412	1.065(0.631-1.799)	0.813
Serum sodium	0.924(0.849-1.005)	0.066	0.954(0.893-1.018)	0.156
Blood ammonia	0.991(0.975-1.007)	0.262	0.993(0.981-1.005)	0.251
INR	4.745(1.966-11.455)	0.001	4.816(2.372-9.777)	<0.001
**Recent antibiotic usage** †	0.768(0.245-2.414)	0.652	0.675(0.279-1.689)	0.401
**ACLF**	4.904(1.776-13.543)	0.002	3.878(1.754-8.575)	0.001
**MELD score**	1.118(0.1084-1.153)	<0.001	1.108(1.082-1.133)	<0.001

†Antibiotic therapy within two weeks before enrollment.

Statistical analysis was performed using univariate Cox proportional hazard models.

Abbreviation: BIs, bacterial infections; GIH, gastrointestinal hemorrhage; HE, hepatic encephalopathy; SBP, spontaneous bacterial peritonitis; CRP, C-reactive protein; HB, hemoglobin; ALB, albumin; ALT, alanine aminotransferase; Cr, creatinine; INR, international standard ratio; ACLF, acute-on-chronic liver failure; MELD score, Model for End-Stage Liver Disease score.
